# Mass Spectrometry Imaging Elucidates the Precise Localization and Site-Specific Functions of Skin Lipids

**DOI:** 10.3390/ijms262412114

**Published:** 2025-12-16

**Authors:** Shown Tokoro, Tadayuki Ogawa, Shujiro Hayashi, Ken Igawa

**Affiliations:** 1 Department of Dermatology, Dokkyo Medical University, 880 Kitakobayashi, Mibu, Shimotsuga, Tochigi 3210293, Japan; shayashi@dokkyomed.ac.jp (S.H.); igawa@dokkyomed.ac.jp (K.I.); 2 Laboratory for Molecular Pathobiology, Research Center for Advanced Medical Sciences, Dokkyo Medical University, 880 Kitakobayashi, Mibu, Shimotsuga, Tochigi 3210293, Japan

**Keywords:** mass spectrometry imaging, skin lipid, intercellular lipid, ceramide, cholesterol, free fatty acid, cholesterol sulfate, phospholipid

## Abstract

Lipids are essential for the skin, playing a crucial role in forming plasma membranes and maintaining the skin’s permeability barrier and hydration. Intercellular lipids fill the spaces between corneocytes and contribute to the barrier function. Lipid abnormalities in the skin have been observed in many skin diseases, including atopic dermatitis and psoriasis. However, the specific localization and roles of skin lipids at particular sites remain incompletely elucidated due to the limited methods available for comprehensive lipid analysis. This study aims to precisely determine the localization of skin lipids, especially intercellular lipids, and investigate their roles and metabolism using mass spectrometry imaging (MSI). We conducted high-resolution (spatial resolution: 5 µm) matrix-assisted laser desorption/ionization (MALDI)-MSI on the lower back and buttocks and created overlay images of skin lipids to clarify their precise localizations. Ceramide was localized in the outermost layer among intercellular lipids. Cholesterol and free fatty acids were present in the stratum corneum but were at trace levels in the outermost layer. Cholesterol sulfate was abundant in the granular layer and gradually decreased in the stratum corneum, promoting desquamation. Phospholipids were confined to the viable epidermis (stratum corneum-/epidermis+), which forms the plasma membrane. A significant increase in mass intensity in the stratum corneum was observed for ceramide, sphingoid base, cholesterol, and free fatty acids, along with a decrease in phospholipids compared with those in the viable epidermis, based on region of interest analysis (Mann–Whitney test, *p* < 0.0005). We clarified the precise localization of skin lipids, particularly intercellular lipids. Our findings supported the reported functions of skin lipids at specific sites. Skin lipids are metabolized to form intercellular lipids in the stratum corneum, which are essential for the skin barrier. Our current lipid localization data serve as a baseline, or healthy control dataset, for future MSI-based lipid biomarker research in disease groups.

## 1. Introduction

The skin serves as a barrier and biosensor against environmental threats and is essential for retaining body water and electrolytes. Lipids play a crucial role in the formation of plasma membranes in the viable epidermis and the skin permeability barrier in the stratum corneum, which are essential for survival [1,2].

The stratum corneum (SC) is the most crucial structure of the skin permeability barrier, composed of corneocytes and intercellular lipids. The intercellular lipids fill the spaces between corneocytes in a “brick and mortar” model, contributing to the barrier function.

Keratinocytes produce filaggrin, loricrin, involucrin, and other proteins during cornification to differentiate into physically robust corneocytes [3], while intercellular lipids are synthesized by the release of precursor lipids and metabolic enzymes stored in the lamellar granules [4,5]. It is noteworthy that the skin permeability barrier is maintained dynamically.

Intercellular lipids consist of ceramide (Cer), cholesterol (Chol), and free fatty acids (FFAs) in a molar ratio of approximately 1:1:1 [6], and more than 1500 species of skin Cer alone have been identified [7]. These intercellular lipids are organized to form complex multilayered lipid bilayers, or lamellar membranes, which are essential for the skin permeability barrier and hydration [8]. Lipid abnormalities in the epidermis have been reported in many skin diseases, including atopic dermatitis and psoriasis [2]. However, the precise localization of each intercellular lipid remains incompletely elucidated due to limited analytical methods for comprehensive lipid analysis.

Mass spectrometry imaging (MSI) is an advanced technology that does not require labeling and allows for the integrated analysis of molecular-specific mass information and the spatial distribution of target molecules using only tissue [9], making it ideally suited for skin lipid analysis due to the ease of sample collection.

In the present study, we visualized Cer and FFAs alongside previously reported lipids [sphingoid base (SB), Chol, Chol sulfate (CS), phospholipid (PL)] [10]. We also created overlay images of skin lipids within the tissue for localization analysis. Finally, we performed region-of-interest (ROI) analysis using the Mann–Whitney test to determine the significance of differences in MS intensity between the stratum corneum and the viable epidermis. Our MSI and ROI results demonstrated the precise localization of skin lipids and supported the reported site-specific functions. Our goal is to establish a detailed baseline lipid map of healthy epidermis, which could serve as a reference for future MSI-based research on lipid biomarkers in disease groups.

## 2. Results

### 2.1. Histological Findings of the Healthy Specimens

**Figure 1:**
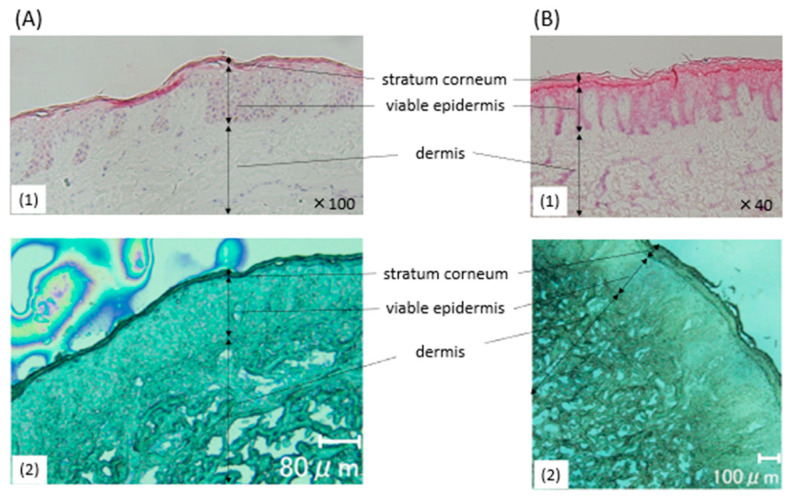
Histological findings of normal skin (NS)1 and NS 2. (**A1**) Histological findings of NS 1 with haematoxylin–eosin staining (HE) of cryopreserved tissue, ×100. (**A2**) 25-dihydroxybenzoic acid (DHB) matrix vapor-deposited NS 1. Bar: 80 μm. (**B1**) Histological findings of NS 2 (HE, cryopreserved tissue), ×40. (**B2**) DHB matrix vapor-deposited NS 2. Bar: 100 μm.

**Figure 2:**
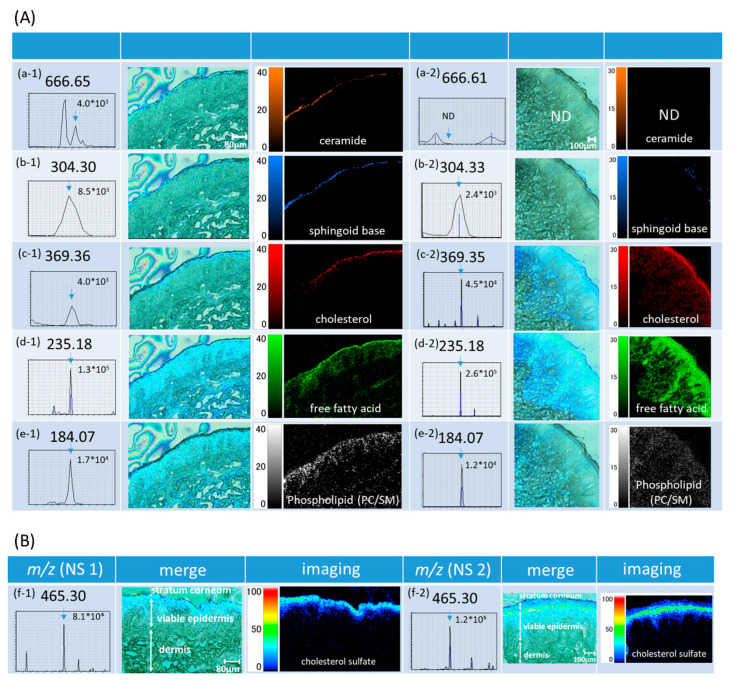
(**A**) Mass Spectrometry Imaging (MSI) of normal skin (NS) 1 (lower back) and NS 2 (buttocks), 25-dihydroxybenzoic acid [DHB] matrix. (**a-1**) NS 1, *m*/*z*: 666.65 [M+H]^+^, ceramide (Cer, orange), (**a-2**) NS 2, *m*/*z*: 666.61 [M+H]^+^, ceramide (Cer, orange), ND: Not detected. (**b-1**) NS 1, *m*/*z*: 304.30 [M+H]^+^, sphingoid base (SB, blue), (**b-2**) NS 2, *m*/*z*: 304.33 [M+H]^+^, sphingoid base (SB, blue), (**c-1**) NS 1, *m*/*z*: 369.36 [M+H]^+^, cholesterol (Chol, red), (**c-2**) NS 2, *m*/*z*: 369.35 [M+H]^+^, cholesterol (Chol, red), (**d-1**) NS 1, *m*/*z*: 235.18 [M+H]^+^, free fatty acid (FFA, green), (**d-2**) NS 2, *m*/*z*: 235.18 [M+H]^+^, free fatty acid (FFA, green), (**e-1**) NS 1, *m*/*z*: 184.07 [M+H]^+^, phospholipid (PC/SM, white), (**e-2**) NS 2, *m*/*z*: 184.07 [M+H]^+^, phospholipid (PC/SM, white), Note, *m*/*z* 184.07 is precisely phosphocholine. Refer to the main text. (**B**) MSI of normal skin (NS) 1 (lower back) and NS2 (buttocks), 9-aminoacridine [9AA] matrix. (**f-1**) NS 1, *m*/*z*: 465.30 [M-H]^−^, cholesterol sulfate (CS, rainbow). (**f-2**) NS 2, *m*/*z*: 465.30 [M-H]^−^, cholesterol sulfate (CS, rainbow).

In the present study, we used surplus skin from the lower back and the buttocks that had undergone excision of epidermal cysts as healthy specimens. The sampled areas were taken from clinically normal skin outside the cyst margins. Histology of the lower back revealed a nearly normal appearance of the stratum corneum, viable epidermis, and dermis [Figure 1(A1)]. Clinically normal skin with mild hyperkeratosis, possibly caused by pressure, was observed on the buttocks [Figure 1(B1)]. Both tissues showed a basket-weave structure in the stratum corneum, indicating a physiological cornification. We conducted MSI after vapor-depositing the DHB matrix [Figure 1(A2,B2), Figure 2(A)] and the 9AA matrix [Figure 2(B)] onto the lower back and buttocks specimens.

### 2.2. MSI of Skin Lipids

MSI demonstrated clear images of skin lipids. Each lipid displayed specific and distinct localizations in particular sites, mainly in the stratum corneum or the viable epidermis (Figure 2).

#### 2.2.1. Lipids in the Stratum Corneum—Intercellular Lipids-

The peak at *m*/*z* 666.65 [Figure 2(Aa-1)], exclusively localized in the SC, was identified as ceramide. We were unable to detect Cer in the buttock specimen [Figure 2(Aa-2)]. The peak at *m*/*z* 304.30 (b), also localized in the SC, was assigned to sphingoid base. The peaks at *m*/*z* 369.36 (c) and *m*/*z* 235.18 (d) were attributed to Chol and FFAs, respectively. Both lipids were highly abundant in the granular layer and the SC but decreased in the outer SC, where Cer predominates. Chol and FFAs were also present in the viable epidermis and dermis, with FFAs being more prevalent.

#### 2.2.2. Cholesterol Sulfate

The peak at *m*/*z* 465.30 [Figure 2(Bf)] was distributed in the upper epidermis using the 9AA matrix, showing the highest concentration in the stratum granulosum and decreasing in the outer SC, where desquamation occurs. *m*/*z* 465.30 was assigned to cholesterol sulfate, displaying a tri-layer pattern known as the “epidermal cholesterol cycle” [11]. A tri-layer pattern of CS was more prominent in the buttock skin [Figure 2(Bf-2)], which has a thicker SC, than in the lower back [Figure 2(Bf-1)], but the localization of CS was the same at both sites.

#### 2.2.3. Lipids in the Viable Epidermis

The peak at *m*/*z* 184.07 [Figure 2(Ae-1,Ae-2)] was localized to the viable epidermis. This molecule was found at high levels in the stratum basale and spinosum, but only at trace levels in the SC. *m*/*z* 184.07 was attributed to the PC headgroup (phosphocholine).

### 2.3. MS/MS for Identification of Cer and Chol

**Figure 3:**
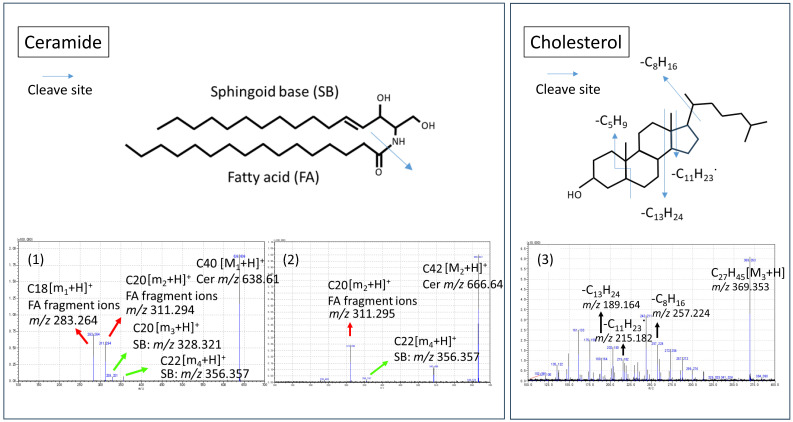
Identification of Ceramide and Cholesterol by MS/MS. (**1**) Product ions of *m*/*z* 638.61 (precursor ion) produced by collision-induced dissociation (CID). (**2**) Product ions of *m*/*z* 666.64 produced by CID. (**3**) Product ions of *m*/*z* 369.36 produced by CID. Red arrows: FA fragment ions. Green arrows: Sphingoid base (SB). Black arrows: Product ions of *m*/*z* 369.35 (Chol). Blue arrows: Cleave site.

MS/MS was performed to identify ceramide and cholesterol (Figure 3). *m*/*z* 638.61 (1) and *m*/*z* 666.64 (2) cleaved into FA fragment ions and SB, the key components of Cer, confirming that they are Cer. Interestingly, Cer at *m*/*z* 638.61(1) produced two pairs of FA fragment ions and SB, (*m*/*z* 311.29, *m*/*z* 328.32) and (*m*/*z* 283.26, *m*/*z* 356.36), respectively, and Cer at *m*/*z* 666.64 (2) also produced FA fragment ions at *m*/*z* 311.29 and SB at *m*/*z* 356.36. These findings infer a “Lego-like” precursor–product relationship in skin lipid metabolism.

The peak at *m*/*z* 396.36 (3) was identified as Chol by matching the fragmentation pattern of Chol [12]. We then generated overlay images of skin lipids using the built-in software for localization analysis.

### 2.4. Localization Analysis of Intercellular Lipids

#### 2.4.1. Ceramide Is Localized in the Outermost Layer Among Intercellular Lipids

**Figure 4:**
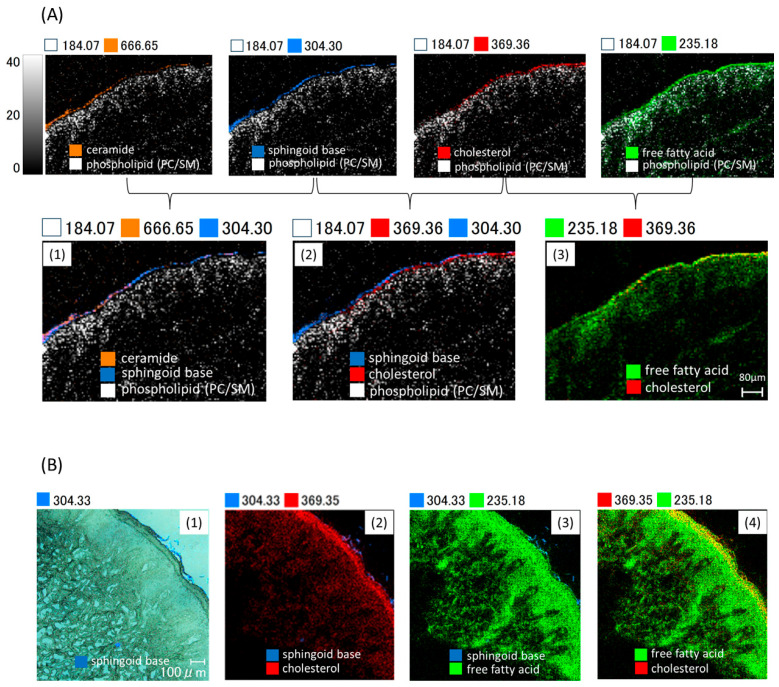
(**A**) Overlay image of normal skin (NS) 1 sample. (**A1**) Overlay image of ceramide (Cer), sphingoid base (SB), and phospholipid (PL). (**A2**) Overlay image of SB, Chol, and PL. (**A3**) Overlay image of free fatty acid (FFA) and Chol. (**B**) Overlay image of NS 2 sample. (**B1**) SB (merge) (**B2**) Overlay image of SB and Chol. (**B3**) Overlay image of SB and FFA. (**B4**) Overlay image of FFA and Chol.

The built-in software for localization analysis created overlay images of Cer, SB, Chol, FFAs, and PLs (Figure 4). The overlay image of skin lipids revealed that Cer and SB coincided in the SC (1) and that Chol and FFAs coincided in the SC (3). Additionally, SB was localized on the outer side of Chol (2), indicating that sphingolipids (Cer, SB) are localized outside of Chol and FFAs.

#### 2.4.2. Sphingoid Base Is Lost Through Desquamation

Desquamation of the outer SC was observed in the buttock specimen [Figure 4(B1)]. Interestingly, detached scales contained abundant SB, indicating that SB is lost through desquamation. Note, SB might be a laser-fragmented form of Cer [refer to Section 3 (2)].

### 2.5. ROI Analysis

We performed an ROI analysis using the Mann–Whitney test to assess the significance of the difference in MS intensity between the stratum corneum and the viable epidermis. The ROI revealed a substantial increase in mass intensities of Cer, SB, Chol, and FFA, along with a decrease in phospholipids (PC/SM) in the stratum corneum, consistent with the MSI findings. These findings indicate that skin lipids are metabolized to form intercellular lipids in the SC. Results are shown in Table 1.

## 3. Discussion

In 2024, our group performed high-resolution MALDI-MSI of human skin and reported lipid changes during keratinization [10]. In the present study, we conducted high-resolution MALDI-MSI and successfully visualized Cer and FFAs alongside previously reported lipids (SB, Chol, CS, PL). The ROI revealed a substantial increase in mass intensities of Cer, SB, Chol, and FFA, along with a decrease in phospholipids (PC/SM) in the stratum corneum, consistent with the MSI findings. Additionally, we generated overlay images of skin lipids, especially intercellular lipids, and clarified the precise localization of each lipid.

**Figure 5:**
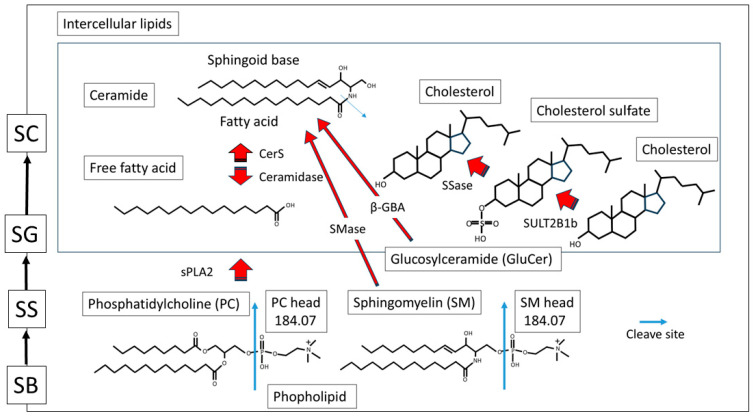
Schema of skin lipid metabolism with chemical structures. Red arrows: Skin lipid metabolism and lipid metabolic enzymes. Blue arrows: Cleave site. CerS, ceramide synthase; sPLA2, secretory phospholipase A2; SMase, sphingomyelinase; β-GBA, β-glucocerebrosidase; SSase, steroid sulfatase; SULT2B1b; sulfotransferase2B1b; SC, stratum corneum; SG, stratum granulosum; SS, stratum spinosum; SB, stratum basale.

Here, we also discuss the roles and metabolism of skin lipids in specific locations. The schema of skin lipid metabolism during keratinization, including chemical structures, is shown below (Figure 5).

(1) Ceramide

The peaks at *m*/*z* 666.65 [Figure 2(Aa-1), Figure 3(2)] and *m*/*z* 638.61 [Figure 3(1)] were identified as Cers by mass spectrometry database and MS/MS analysis. Both peaks cleaved into FA fragment ions and SB, the key components of Cer, by collision-induced dissociation (Figure 3). Cer is the primary lipid responsible for skin barrier function and hydration. In this study, we demonstrated that Cer localizes to the outermost layer among intercellular lipids [Figure 4(A)]. Cer contains long-chain fatty acids and is highly hydrophobic, which makes its position at the outermost layer logical.

Human SC is reported to be acidic at pH 4.5–5.5 [13,14], and β-glucocerebrosidase (β-GBA) and acidic sphingomyelinase (SMase), which synthesize Cer from glucosylceramide (GluCer) and sphingomyelin (SM) (Figure 5), are activated by an acidic pH of 5.6 and 4.5, respectively [15].

Recently, Fukuda et al. [16] reported a tri-layered pH profile in murine SC. These findings indicate that Cer synthesis occurs only in the outer SC at an optimal acidic pH. Intriguingly, we observed the loss of SB (possibly laser-fragmented Cer) via desquamation (Figure 4B), suggesting that Cer is continuously lost via desquamation and is resynthesized in the outer SC at an optimal pH to compensate for these losses. However, in murine outer SC, pH ranges from acidic to neutral, which is unsuitable for activating β-GBA and acidic SMase in human skin. Therefore, further detailed research is necessary to clarify the relationship between human layer-specific pH and enzyme activity in SC.

(2) Sphingoid Base

The peak at *m*/*z* 304.30 matched only SB in the lipid mass spectrometry database. Therefore, the peak at *m*/*z* 304.30 [Figure 2(Ab)] was assigned to SB. An overlay image of SB and Cer demonstrated identical localizations in the outer SC [Figure 4(A1)]. Since SB is the main component of Cer, we speculated that SB might be a laser-fragmented form of Cer. Although MALDI-MSI cannot reliably distinguish free SB from fragment-derived SB signals, including *m*/*z* 304.30, the SB signal in MSI likely reflects a fragment-derived species rather than a separately localized pool of free SB.

(3) Cholesterol

The peak at *m*/*z* 369.35 [Figure 2(Ac)] was identified as cholesterol through MS/MS analysis [Figure 3(3)]. The overlay image of Chol and FFA demonstrated identical distributions in the SC. Cer, Chol, and FFA are the primary components of intercellular lipids forming the lamellar membrane between the corneocytes. However, Chol and FFA decreased in the outer SC, and the overlay image of Chol and Cer exhibited separate distributions. Chol was also found in the viable epidermis and dermis at low levels.

(4) Free fatty acids

The peak at *m*/*z* 235.18 matched only FFA in the lipid mass spectrometry database, and the distribution aligned with that of reported FFA [17]. Therefore, the peak at *m*/*z* 235.18 [Figure 2(Ad)] was assigned to FFA. However, it is important to note that MALDI-MSI cannot reliably distinguish true FFAs from fragment-derived FA signals, including *m*/*z* 235.18. Chol and FFA showed identical distributions in the SC, as previously stated. FFAs were also found in the viable epidermis and dermis, with higher mass intensities than Chol.

(5) Cholesterol Sulfate

The peak at *m*/*z* 465.30 [Figure 2(Bf)] was identified as CS through a mass spectrometry database and the characteristic distribution reported in past documents. CS is a serine protease inhibitor that plays a crucial role in the process of desquamation. In healthy skin, SULT2B1b sulfates Chol in the upper epidermis, and CS is most abundant in the SG. However, CS levels decrease in the SC as it is desulfurized by steroid sulfatase (SSase) [11,18] (Figure 5). In 1984, Epstein et al. described these changes and termed them “the epidermal cholesterol sulfate cycle”. The desulfurization of CS in the SC by SSase activates proteases such as kallikrein, which promotes desquamation [11,18]. In X-linked ichthyosis, where SSase is genetically defective, CS accumulates in the SC, leading to delayed desquamation and ichthyosis [18].

(6) Phospholipid (PC/SM) (*m*/*z* 184.07)

The peak at *m*/*z* 184.07 [Figure 2(Ae)] was assigned to the PC headgroup, or laser-fragmented phosphocholine, by cross-checking in both the previous literature [19] and the NIH mass spectrometry database. Phosphocholine was considered to originate from the cell membrane. However, SM, another component of the phospholipid bilayer, also contains phosphocholine in its structure (Figure 5). Therefore, *m*/*z* 184.07 represents phosphocholine-containing lipids (PC/SM), and is labeled as phospholipid (PC/SM) in the text and figures.

Keratinocyte cell membranes and organelles are composed of phospholipid bilayers, with PC as the dominant component [2]. PL levels decreased in the SG and were at trace levels in the SC [Figure 2(Ae)]. The phospholipid bilayer of keratinocyte cell membranes undergoes alterations, including the loss of membrane lipids to form the cornified envelope and the outer cornified lipid envelope during cornification [6]. This cornification process explains the reduction and depletion of PL observed in the SC.

In 2024, Matsui et al. [20] reported “corneoptosis,” a specialized form of keratinocyte death. They noted that an influx of intracellular Ca^+^ and subsequent cell acidification at SG1 result in “corneoptosis” and the subsequent loss of the nuclear membrane, DNA, and organelles [21]. Based on their findings, we speculated that “corneoptosis” also contributes to the loss of PL in the SC.

While keratinocytes differentiate into corneocytes and serve as a physical barrier in the SC, skin lipids are metabolized to form intercellular lipids, with Cer localized at the outermost layer. Although the relevance is unclear, it is highly intriguing that the viable epidermis is rich in PL, especially phosphatidylcholine, which forms the plasma membranes of “individual cells.” Meanwhile, the stratum corneum contains high levels of intercellular lipids, particularly ceramide, which is crucial for the barrier function of the “whole body.” Skin lipids and corneocytes conclude their roles through desquamation due to the decrease in cholesterol sulfate.

## 4. Conclusions

Our MSI findings elucidated the precise localization of skin lipids. Each lipid plays a specific role in certain sites, forming a “skin lipid orchestra” that maintains skin homeostasis. Functional roles for lipids are inferred from the literature, and more detailed, site-specific functional validation of skin lipids is necessary in the future. The current MSI-based data on lipid localization in healthy skin provides a foundation for future MSI-based lipid biomarker research in disease groups.

## 5. Limitations

Lipids are diverse, with more than 1500 species identified for Cer alone. Therefore, MSI results may differ depending on acyl chain length, isotopic composition, and isomer composition. In this study, we performed MSI in a setting suitable for detecting a wide range of lipids, but not for detecting high-molecular-weight molecules, including acylceramides. Additionally, this study serves as a proof-of-concept with a small sample size, and larger, multi-site validation will be necessary in the future. Despite careful identification of the detected molecules, the possibility of misidentification cannot be ruled out.

## 6. Materials and Methods

### 6.1. Skin Samples

The skin subjects were recruited from outpatients at the Department of Dermatology, Dokkyo Medical University, and provided written informed consent to participate in this study. This research was approved by the Ethics Review Board of Dokkyo Medical University (Ethics Application Number: R-62-5J). Surplus skin samples (NS 1: lower back, NS 2: buttocks) that underwent excision of epidermal cysts were used for imaging. The sampled areas were taken from clinically normal skin outside the cyst margins and were removed as part of the standard surgical procedure.

### 6.2. Materials

9-Aminoacridine (9AA, Merck, Darmstadt, Germany) and 25-dihydroxybenzoic acid (DHB, Sigma–Aldrich, St. Louis, MO, USA) were purchased for use as matrices. Carboxymethyl cellulose sodium salt 4% (FUJIFILM Wako Pure Chemical Corporation, Osaka, Japan) was used for embedding samples for sectioning and imaging. A cryostat (CM3050S, Leica, Wetzlar, Germany) was used to cut the frozen skin samples into 7-μm slices. Indium tin oxide glass slides were purchased from Matsunami Glass (Bellingham, WA, USA) for MSI. Tissues were stained by haematoxylin–eosin (HE) for histological observations.

### 6.3. Matrix-Assisted Laser Desorption/Ionization-Mass Spectrometry Imaging (MALDI-MSI)

MALDI-MSI was performed on 2 cases of skin samples from different regions. An iMScope (Trio, Shimadzu, Kyoto, Japan) was used for molecular imaging and lipid identification. iMScope converts the laser-ionized molecular information into a mass spectrum with mass-to-charge ratios (*m*/*z*) using a built-in time-of-flight mass spectrometer. The localization of analyte molecules was visualized by integrating the mass spectral data of each irradiated spot. We used a 9AA matrix for the ionization of CS and a DHB matrix for the ionization of other lipids. The laser was set to a 5 μm diameter and an intensity of 15.2 J (laser wavelength: 355 nm, pulse duration < 7.5 ns, laser shot count: 50, repetition rate: 1000 Hz). MSI was performed with a spatial resolution of 5 μm in positive ion mode and 5 or 10 μm in negative ion mode. Non-target MALDI-MSI was conducted using DHB and 9AA matrices, suitable for lipid ionization. The molecules ionized by irradiation vary depending on the matrix selected. Positive ions ([M+H]^+^) and negative ions ([M-H]^−^) were produced by DHB and 9AA matrices, respectively. A wide range of MS spectra was detected under the current settings, but only a limited number of molecules were clearly visualized and localized in the skin tissue using the iMScope, which we focused on.

### 6.4. Identification of Lipids

We first attempted to identify the detected molecules by cross-checking their *m*/*z* values against previously published literature and the NIH Mass Spectrometry Database “https://www.metabolomicsworkbench.org (accessed on 1 January 2021)”. We searched for matches for the *m*/*z* ([M+H]^+^) detected with the DHB matrix and the *m*/*z* ([M-H]^−^) detected with the 9AA matrix in the MS Spectrometry Database for molecular identification. Then, we performed MS/MS analysis to confirm molecular identities. Product ions produced by collision-induced dissociation (CID) were analyzed to determine precursor ions. Eventually, we identified Cer, SB, Chol, FFA, PL (PC/SM), and CS with distinct localization.

### 6.5. Localization Analyses of Skin Lipids

Optical images of Cer, SB, Chol, FFA, PL (PC/SM), and CS were acquired by the optical microscope built into iMScope, and MSI and optical layers were accurately aligned manually with the built-in XY-axis adjustment device. Built-in localization analysis software (Imaging MS Solution, Shimadzu, Kyoto, Japan) was used to generate overlay images of skin lipids for localization analysis.

### 6.6. Region of Interest (ROI) Analysis

Statistical comparisons of MS signal intensities between ROI A (stratum corneum) and ROI B (viable epidermis) were performed using the Mann–Whitney U test (including *p*-value, mean, standard deviation, and median) due to the non-normal distribution of the data. All analyses were performed with built-in ROI analysis software (Imaging MS Solution, Shimadzu, Kyoto, Japan). We analyzed ROI for NS1 and NS2 separately. NS1 contained 457 spectra in the SC region and 2850 in the viable epidermis. NS2 included 3095 spectra in the SC region and 9300 in the viable epidermis.

## Figures and Tables

**Table 1:** Region-of-interest (ROI) analysis of NS1 and NS2 specimens. (ROI A: stratum corneum vs. ROI B: viable epidermis).

NS1		ROI A: Stratum Corneum			ROI B: Viable Epidermis		
*m*/*z*	*p*-value	mean	standard deviation	median	mean	standard deviation	median
184.07	5.8 × 10^−19^	21.6	46.7	0	54.9	80.2	0
235.18	3.0 × 10^−73^	640.6	472.6	540.0	267.5	214.0	229.5
304.30	0	178.5	267.2	0	0.2	3.7	0
369.35	7.8 × 10^−161^	77.0	95.1	75.0	6.8	26.1	0
666.64	3.2 × 10^−173^	72.0	125.5	0	1.5	11.6	0
NS2		ROI A: Stratum corneum			ROI B: Viable epidermis		
*m*/*z*	*p*-value	mean	standard deviation	median	mean	standard deviation	median
184.09	4.5 × 10^−66^	10.2	28.5	0	24.3	44.0	0
235.17	3.2 × 10^−9^	637.7	357.6	592.0	590.5	320.8	542.0
304.30	7.6 × 10^−21^	18.7	76.8	0	0.2	3.4	0
369.34	0	203.7	147.6	180.0	95.6	80.9	78.0

## Data Availability

The data that support the findings of this study are available from the corresponding author upon reasonable request.

## Abbreviations

The following abbreviations are used in this manuscript:

Cer	ceramide
SB	sphingoid base
Chol	cholesterol
FFA	free fatty acid
PC	phosphatidylcholine
PL	phospholipid
SM	sphingomyelin
CS	cholesterol sulfate
MSI	mass spectrometry imaging
NS	normal skin
SC	stratum corneum
